# Investigation of Platelet Function Analyzer 200 platelet function measurements in healthy cats and cats receiving clopidogrel

**DOI:** 10.1177/10406387231197440

**Published:** 2023-08-30

**Authors:** Matthew R. Kornya, Anthony C. G. Abrams-Ogg, Shauna L. Blois, R. Darren Wood

**Affiliations:** Departments of Clinical Studies, Ontario Veterinary College, University of Guelph, Guelph, Ontario, Canada; Departments of Clinical Studies, Ontario Veterinary College, University of Guelph, Guelph, Ontario, Canada; Departments of Clinical Studies, Ontario Veterinary College, University of Guelph, Guelph, Ontario, Canada; Pathobiology, Ontario Veterinary College, University of Guelph, Guelph, Ontario, Canada

**Keywords:** antithrombotic, clopidogrel, platelet function

## Abstract

The Platelet Function Analyzer 200 (PFA-200; Siemens) is an in vitro substitute for in vivo bleeding time that is designed to investigate platelet function in a more physiologic manner than traditional aggregometry. The analyzer reports a closure time (CT) as a marker of platelet function, and may also report the calculated platelet function measurement primary hemostasis components, PHC1 and PHC2. These incorporate the measured total volume (TV) of blood aspirated and the initial flow rate (IF). We determined, for the COL/ADP and P2Y cartridges, the median total volume (TV_median_), and RIs for CT, IF, TV, PHC1, and PHC2, and investigated the sensitivity and specificity of those parameters at the determined interpretation thresholds in determination of the clopidogrel effect. Healthy client-owned cats were recruited prospectively to determine RIs for CT, IF, TV, PHC1, and PHC2. Healthy blood-donor cats and cats on clopidogrel therapy were included retrospectively to determine test performance. In 20 healthy cats, RIs for COL/ADP were CT (19.5–87.2 s), IF (199–278 µL/min), TV (199–332 µL), PHC1 (94–106%), and PHC2 (52–148%); and for P2Y, CT (4.2–94.3 s), IF (112–208 µL/min), TV (151–294 µL), PHC1 (35–178%), and PHC2 (90–109%). CVs were calculated for all of these values. Specificity for detection of the clopidogrel effect was calculated from a group of healthy blood donors, and sensitivity for detection of the clopidogrel effect from a group of cats with known clopidogrel effect. Sensitivity and specificity were, for COL/ADP: CT (83.3%, 66.6%), IF (41.4%, 83.3%), TV (83.3%, 100%), PHC1 (100%, 100%) and PHC2 (100%, 83.3%); and for P2Y: CT (100%, 94.4%), IF (30%, 44.4%), TV (100%, 94.4%), PHC1 (100%, 100%), and PHC2 (100%, 97.7%). These PFA-200 values may be beneficial in the determination of platelet function in cats.

The Innovance Platelet Function Analyzer 200 (PFA-200; Siemens) and its predecessor, the PFA-100, are hemostasis analyzers designed to evaluate platelet function in a more physiologic manner than traditional aggregometry-based methods.^11^ The PFA test has been compared to an in vitro correlate of the buccal mucosal bleeding time.^21^ Several test cartridges are available for the PFA, including the original collagen/adenosine 5’-diphosphate (COL/ADP) and collagen/epinephrine cartridges, and the newer P2Y cartridge, which has been optimized to detect the clopidogrel effect.^24^

Although there are many potential uses of the PFA in detecting congenital (e.g., von Willebrand disease, Glanz-mann thrombasthenia) and acquired defects in platelet function,^32^ the results indicate only a defect in platelet function and are not specific for a given condition.^5^ One role of the PFA in human and veterinary medicine is to confirm adequate clopidogrel effect in patients receiving antithrombotic therapy,^14,24^ given that insufficient clopidogrel effect, referred to as clopidogrel resistance or high-on-treatment platelet reactivity, has been reported in humans, dogs, and cats.^2,3,10,29^ Determining which cats are resistant to clopidogrel allows dose adjustment or initiation of alternative medications. A consensus on the best monitoring protocol has not been reached, but it has been suggested that a combination of platelet function tests may be ideal.^25^ Identifying non-responders is vital because the first clinical sign of platelet dysfunction is usually thromboembolism,^19^ which has a reported fatality rate in cats of ~50%.^1,7,15,27^ Thromboembolism does not necessarily imply clopidogrel failure, given that multiple pathways may be involved in clot generation.^10^ When thrombosis occurs during clopidogrel therapy, it is important to determine if a dose adjustment, drug class change, or addition of another drug is indicated.

Two cartridges are available for the PFA to detect the clopidogrel effect, COL/ADP and P2Y, which may have different sensitivity and specificity.^24^ Given that the COL/ADP and P2Y cartridges generate different RIs, direct comparison of results from the 2 cartridges is difficult.^22^ As well, interpretation of the output “closure time” (CT) value may not be intuitive to practitioners ordering tests who lack familiarity with the analyzer and its RIs. A body of human psychometric data suggests that people have different and perhaps superior comprehension of values expressed as a percentage change compared to absolute changes.^26,31^ Finally, the CT value accounts only for the time taken for the test to terminate and does not incorporate other potentially significant parameters such as the initial flow rate (IF) of blood and the total volume (TV) of blood aspirated. The significance of these parameters, and whether their inclusion may be helpful in analysis, is not completely known, although in canine medicine the TV has been reported in a single paper to not be useful.^18^

To standardize CT values for direct comparison and account for several of the other variables produced by the PFA, the primary hemostasis components 1 (PHC1) and 2 (PHC2) were developed by the manufacturer. These are available based on platelet function measurements that are non-standardly reported values available on the analyzer. Their method of calculation has been described^22^; however, they have not been reported in the human or veterinary literature, to our knowledge. Given that these parameters express platelet function as a percentage of “normal,” they may be more readily intuitive to those unfamiliar with the analyzer. To determine these values, CT is not used, but rather combinations of IF and TV are used. For the purpose of normalizing the PHC parameters to 100%, values for expected TV in healthy individuals must be determined. The patent describing these parameters defines median TV (TV_median_) for humans based on an unreported sample size and does not report a range; however, given that cats are generally considered to have more reactive platelets than humans,^7,23^ values would be expected to be different.

The other parameter required to calculate PHC is the dead volume (DV) of a cartridge. This is the volume of air aspirated before blood flow occurs and is a fixed volume for each cartridge type.^22^ This is not a patient-dependent parameter, and median values reported by the manufacturer for the cartridges may be used.

PHC1 is a relatively simple parameter calculated as a ratio of the TV in the sample compared to the reference TV_median_ with the following equation:

$$\begin{array}{c} PHC1\;=\;\left(TV_{median}-\;DV_{median}\right)\;\\ \;\;\;\times \;100\%/\left(TV_{sample}-\;DV_{median}\right). \end{array}$$

PHC2 is a more complex parameter that is calculated by multiplying the IF of a sample by 5 min (300 s), which is the maximum time a sample will be analyzed. This determines the theoretical maximum volume aspirated in a subject with no platelet function. The sample’s TV is subtracted from this theoretical maximal volume to give a value for the volume of blood that could have been, but was not, aspirated. This is repeated using the reference median TV and the values expressed as a ratio. Although more complex than PHC1, the incorporation of IF in PHC2 corrects for some of the variability in PHC1 and gives numbers in a tighter range above and below 100%. PHC2 is calculated as:

$$\begin{array}{c} PHC2\;=\;\left(\begin{array}{l} 5\;min\times IF_{sample}\\ -\;\left[TV_{median}-\;DV_{median}\right] \end{array}\right)\;\\ \;\;\;\times \;100\%/\left(\begin{array}{l} 5\;min\;\times \;IF_{sample}\\ -\;\left[TV_{sample}-\;DV_{median}\right] \end{array}\right). \end{array}$$

We determined the TV_median_ for a group of healthy cats to allow calculation of the PHC1 and PHC2 parameters, to determine the RIs for CT, IF, TV, PHC1, and PHC2, and investigated the utility of these parameters in determining the clopidogrel effect.

## Material and methods

We calculated the RIs for CT, IF, TV, PHC1, and PHC2 from a group of 20 healthy client-owned cats using both the COL/ADP and P2Y cartridges. Note that for PHC2, we inverted the equation as described in the Discussion. The specificity of these tests for detection of the clopidogrel effect was determined by performing these calculations on a group of healthy blood-donor cats (true-negative was considered a result within the RIs), and the sensitivity was determined on a group of cats with known clopidogrel effect (true-positive was considered a result below the lower limit of the RI for PHC1 and PHC2, and above the upper limit of the RI for CT, IF, and TV).

Twenty healthy cats were recruited on a volunteer basis from students, faculty, and staff of a veterinary teaching hospital (“healthy” group). They were confirmed to be healthy based on medical history, physical examination, and routine bloodwork (CBC, serum biochemistry profile, and FeLV/FIV testing), and were excluded if they were receiving any medications known to affect coagulation or platelet function within 1 mo before testing.

Cats were 1–12-y-old (median: 4.5 y). Breed distribution was: 14 domestic shorthairs, 2 domestic longhairs, 1 domestic medium-hair, 1 Persian, 1 Russian Blue, and 1 Devon Rex. Ten cats were castrated males and 10 cats spayed females. Median weight was 4.87 kg (range: 2.65–7.55 kg), median body condition score was 5 of 9 (range: 3–8), and all muscle condition scores were 3 of 3.

Cats received gabapentin PO a median of 2.5 h prior to phlebotomy. Doses were 19.9–37.7 mg/kg (median: 26.5 mg/kg). The skin overlying both jugular veins was clipped, and 4% lidocaine gel (Maxilene 4; Ferndale Laboratories) was applied and covered with a bandage for a minimum of 30 min. The median time from gabapentin administration to local anesthetic application was 72 min (range: 15–255 min), and the median time from local anesthesia to phlebotomy was 76 min (range: 20–95 min). Cats that remained uncooperative and in which venipuncture without excessive platelet activation would have been difficult were administered butorphanol IV (0.2–0.4 mg/kg, Torbugesic; Zoetis) via the medial saphenous vein.

Bandages covering the jugular veins were removed, and the skin was cleansed with isopropyl alcohol and allowed to dry. Cats were gently restrained manually, and jugular venipuncture was performed with a 22-g, 2.5-cm needle attached to a 6-mL syringe. Venipuncture and blood draw were subjectively graded by an observer using a scoring scheme (briefly, initial venipuncture was scored as: 1 = no redirection, immediate flow; 2 = 1–2 redirections; or 3 = multiple redirections and unsuccessful aspiration. Blood flow was graded as: 1 = no stoppage of flow, smooth withdrawal; 2 = one brief flow interruption; or 3 = multiple interruptions or redirection after flow started). Samples that were considered insufficient quality (> 2 for either venipuncture or flow) were discarded and venipuncture repeated with the opposite jugular vein. All samples were collected over a 2-wk period.

Samples were immediately transferred to two 1.8-mL 3.2% sodium citrate tubes (Becton Dickinson) and one 1.0-mL 7.5% K_3_-EDTA tube (Plateletworks; Helena Laboratories). Lids were removed from the tubes, the needle removed from the syringe, and the citrate tubes filled by dispensing to the indicator line, followed by filling of the EDTA tube to the indicator line. Citrate tubes designated for P2Y testing were filled first, followed by filling of the tubes designated for COL/ADP testing. Samples in all tubes were mixed with 15 full inversions. Slides were prepared from each EDTA sample and evaluated for platelet clumping. EDTA samples were transported to an on-site first-opinion healthcare hospital (Smith Lane Animal Hospital, Ontario Veterinary College, University of Guelph, Guelph, ON, Canada) for analysis on a ProCyte Dx (Idexx) in duplicate to confirm adequate hematocrit, platelet counts, and no or minimal platelet clumping (i.e., 0–2 small clumps [< 5 platelets per clump]) present on the slide with no apparent effect on platelet count based on platelet estimate from slide approximating analyzer value.

Citrate samples were stored at room temperature for a median of 69 min (range: 52–118 min) before the first analysis. All samples were analyzed in duplicate, beginning with the P2Y samples, followed by the COL/ADP samples. Median time between the first and second P2Y analysis was 9 min (range: 6.4–28 min), between the second P2Y and first COL/ADP analysis 10 min (range: 9.5–23 min), and between the first and second COL/ADP analysis 10 min (range: 8–17 min). If either the first or duplicate analysis had a flow obstruction, a third sample was analyzed after all other analyses were complete, assuming adequate sample volume remained. If the triplicate sample flow was obstructed or an adequate sample was not available, a single data point was recorded for that patient. Median time from collection of blood to completion of analysis was 104 min (range: 84–140 min). Each measurand (CT, IF, TV, PHC1, PHC2) was obtained for both the P2Y and COL/ADP cartridges.

In addition, over 5 y (October 2016–July 2021), up to the enrollment of the previously described cats, PFA-200 testing was performed on 39 healthy blood-donor cats (“blood donor” group). This group of cats was utilized as a retrospective cohort to verify the specificity of the derived RIs in the determination of the clopidogrel effect. True-negative was considered a result within the RI (Suppl. Table 1). Collection procedures from these cats differed from the above cohort; blood was collected under general anesthesia. These cats were healthy based on physical examination, FeLV/FIV tests, and annual health screening (CBC, serum biochemistry profile, ± urinalysis). Thirty cats were castrated males and 9 were spayed females. The median age was 3.4 y (range: 1.0–6.2 y). Thirty-five cats were domestic shorthairs and 4 were domestic longhairs. Anesthesia for blood collection used various injectable drug protocols, and cats were administered oxygen delivered by face mask. The left or right jugular vein was clipped and aseptically prepared, and samples were collected via jugular venipuncture using a 19-ga butterfly needle and 3-mL syringe. The samples for PFA-200 testing were collected prior to the donated sample, aliquoted into 3.2% sodium citrate tubes, and analyzed within 30–180 min of collection (exact times are not available).

Retrospective data were also collected from 44 cats undergoing PFA-200 testing for clopidogrel monitoring from October 2014 to July 2021 (“clopidogrel” group). This group of cats was utilized to verify the sensitivity of values outside the derived RIs for detection of the clopidogrel effect. True-positive was considered below the lower limit of the RI for PHC1 and PHC2, and above the upper limit of the RI for CT, IF, and TV. The cats had been prescribed clopidogrel for a variety of diseases. All cats had been treated for at least 7 d with 18.75 mg of clopidogrel PO q24h at the time of testing. Ten cats were excluded because of incompletely recorded data, and 10 cats were excluded because of flow obstructions preventing accurate interpretation. Of the 24 remaining cats, 3 had CT values within the RIs for normal cats, consistent with clopidogrel resistance (confirmed by lack of clopidogrel effect on a platelet counting–based method [Plateletworks] when performed),^12–14^ leaving 21 cats in the clopidogrel-responsive group. Only data from these 21 cats were utilized. The removal of the 3 cats without clopidogrel effect was necessary to assess sensitivity. These cats all had a lack of clopidogrel effect demonstrated with the Plateletworks assay and a CT within the calculated RI.^12–14^

There were 16 castrated male and 5 spayed female cats, 1–16-y-old. Eleven cats were receiving clopidogrel for primary or secondary prevention of thromboembolism related to cardiomyopathy, 1 cat because of previous disseminated intravascular coagulation, 3 because of ischemic cere-brovascular events, 2 because of pancreatitis, 1 because of phenobarbital-induced pseudolymphoma, 1 because of lymphoma, and 2 because of immune-mediated hematologic disorders. The samples for PFA-200 testing were collected using the same techniques as for healthy client-owned cats and were analyzed within 30–240 min of blood collection.

R, R-Studio, and Microsoft Excel were used for statistical analysis. RIs were calculated with the Excel Add-On Reference Value Advisor using the Anderson–Darling test for normality.^8^ When duplicate results were available, the mean of both results was used for analysis. CVs were calculated for all duplicate data and the means of these CVs calculated. Given that the TV values were not normally distributed, the median value was used as the TV_median_ for determination of PHC parameters. RIs were determined using Reference Value Advisor^8^ using the robust method with or without Box–Cox transformation of the data A Tukey test was used to inspect each dataset for outliers prior to analysis. Two-by-two tables were manually constructed and used to calculate the sensitivity (from cats with known clopidogrel effect) and specificity (from healthy blood donors) of all measurands (CT, IF, TV, PHC1, PHC2) for the determination of the clopidogrel effect based on values above (CT, IF, TV) or below (PHC1, PHC2) the RI determined in healthy client-owned cats.

## Results

We included all 20 healthy client-owned cats in our study. For the samples utilized, 16 of 20 venipunctures were considered good quality (score 1) and 4 of 20 moderate quality (score 2); 16 of 20 blood draws were considered good quality (score 1) and 4 of 20 moderate quality (score 2).

Platelet counts were 159–367 × 10^9^/µL, with a median of 290 × 10^9^/µL. No sample was found to have more than minimal platelet clumping (i.e., 0–2 small clumps present on the slide with no apparent effect on platelet count).

When analyzing the 20 healthy client-owned cats with the COL/ADP cartridge, flow obstruction occurred in 4 of 20 cats, but the triplicate sample did not flow obstruct in any case, therefore duplicate values were available for all cats. For the P2Y cartridge, flow obstructions occurred in 8 of 20 cats; in 3 of 8 cats the triplicate sample also flow obstructed, therefore only a single data point was available for analysis. For the COL/ADP cartridge, all 20 cats had 2 measurements available. For the P2Y cartridge, all cats had at least one measurement available; 17 had 2 measurements available (Table 1).

**Table 1.** CVs for COL/ADP analyses for 20 healthy client-owned cats on the PFA-200. For P2Y analyses, in 17 of the 20 cats, only a single analysis was possible.

Parameter	Median CV % (*n*)
COL/ADP	
CT	9 (20)
IF	9 (20)
TV	4 (20)
PHC1	9 (20)
PHC2	1 (20)
P2Y	
CT	14 (17)
IF	4 (17)
TV	7 (17)
PHC1	21 (17)
PHC2	2 (17)

CT = closure time; IF = initial flow rate; PHC1, PHC2 = primary hemostasis component 1 and 2, respectively; TV = total volume.

For the blood-donor cats, 6 samples were available for COL/ADP (no samples analyzed in duplicate) and 35 samples for P2Y (24 analyzed in duplicate). Many blood-donor cats had several PFA-200 analyses performed during the study period; however, we have included only the first analysis.

TV_median_ for the 20 healthy cats was 266 μL for the COL/ADP cartridge and 216 μL for the P2Y cartridge. Using this value, PHC1 and PHC2 values were calculated for healthy client-owned cats, blood donors, and clopidogrel-responsive cats. CVs were determined for each of the above values (Table 1). RIs were generated for each of the platelet function measurements as well as CT. No outliers were noted; therefore, all cats were included (Table 2).

**Table 2.** RI data of PFA-200 additional platelet function measurements with COL/ADP and P2Y cartridges for 20 healthy client-owned cats.

Measurand	x̄	SD	Median	Min.	Max.	Anderson–Darling *p*-value	Dist.	Method	LRL of RI	URL of RI	90% CI of LRL	90% CI of URL
COL/ADP												
CT, s	56	15	52	4	105	0.026	G	R	20	87	5–34	69–102
IF, μL/s	238	18	24	199	265	231	NG	RT	200	278	187–216	267–291
TV, μL	272	31	266	241	367	0.013	G	R	199	332	164–225	298–359
PHC1, %	99	22	94	52	131	217	NG	RT	94	106	91–97	103–108
PHC2, %	99	3	99	91	103	0.042	G	R	52	147	41–69	131–163
P2Y												
CT, s	58	20	51	36	101	<0.0001	G	R	4	94	–5 to 25	75–113
IF, μL/s	164	22	166	110	211	0.669	NG	RT	112	208	90–133	195–219
TV, μL	223	34	216	182	320	0.084	NG	RT	151	295	129–176	272–317
PHC1, %	95	34	88	38	175	0.830	NG	RT	35	178	26–49	145–211
PHC2, %	98	4	99	87	106	0.127	NG	RT	90	109	86–94	105–112

CT = closure time; Dist. = distribution; G = Gaussian; IF = initial flow rate; LRL = lower reference limit; NG = non-Gaussian; PHC1, PHC2 = primary hemostasis component 1 and 2, respectively; R = robust; RT = robust transformed; TV = total volume; URL = upper reference limit.

Twenty-one cats with clopidogrel effect were enrolled; 12 of the included 21 cats had testing with COL/ADP, only one of which was in duplicate. All 21 included cats had tests performed with the P2Y cartridge, of which 6 were in duplicate.

Sensitivity and specificity of each measurand were calculated (Table 3) for detection of the clopidogrel effect.

**Table 3.** Sensitivity of additional platelet function measurements for the determination of the clopidogrel effect in cats based on a population of successfully clopidogrel-treated cats (*n* = 12 for COL/ADP; *n* = 21 for P2Y), and specificity of the clopidogrel effect in cats based on a population of blood-donor cats (*n* = 6 for COL/ADP; *n* = 35 for P2Y), determined in comparison to a RI derived from 20 healthy cats. True-positive for clopidogrel effect was considered below the lower limit of the RI for PHC1 and PHC2, and above the upper limit of the RI for CT, IF, and TV. True-negative for the clopidogrel effect was considered as a result within RI.

Measurand	Sensitivity, %	Specificity, %
COL/ADP		
CT	83.3	66.6
IF	41.4	83.3
TV	83.3	100
PHC1	100	100
PHC2	100	83.3
P2Y		
CT	100	94.4
IF	30.0	44.4
TV	100	94.4
PHC1	100	100
PHC2	100	97.2

CT = closure time; IF = initial flow rate; PHC1, PHC2 = primary hemostasis component 1 and 2, respectively; TV = total volume.

## Discussion

We investigated the use of the 2 standard platelet function measurements (IF and TV) and 2 novel markers of platelet function (PHC1 and PHC2) on the PFA. The novel markers were described by the manufacturer and are available as additional platelet function measurements; however, they are not routinely produced as output, and little information on these parameters has been reported. We retrieved no reports of these parameters, other than the cited patent, in a search of Google, Google Scholar, PubMed, and Web of Science, suggesting that these have not been investigated previously in cats or dogs. Our study was performed on a PFA-200, which reports the platelet function measurements IF and TV. These are not reported by the older PFA-100 by default; however, they may be activated with specific software from the manufacturer. Likewise, the P2Y cartridge cannot be analyzed on the PFA-100 by default, but this capability may be activated with a manufacturer upgrade.

In the patent describing the PHC1 and PHC2, the equation for PHC2 appears to be discordant in the values used for the numerator and denominator compared to the description of its intent.^22^ The description of the parameter, with normal being theoretically 100% and subjects with platelet dysfunction being < 100%, and the ability for PHC2 to increase and decrease proportionally to both platelet reactivity and PHC1, suggests that PHC2 should be calculated with TV_sample_ in the numerator and TV_median_ in the denominator, and this is the calculation that we used.

TV_median_ for cats was determined to be different from the values reported in humans. The human values for TV_median_ have been reported to be 300 µL for COL/ADP and 223 µL for P2Y^22^; in cats, the values were 266 μL and 216 μL, respectively. This is likely the result of increased platelet reactivity in cats compared to humans, leading to closure or flow obstruction after a smaller volume is aspirated.

CT, TV, PHC1, and PHC2 all showed acceptable sensitivity and specificity for the determination of the clopidogrel effect using both cartridges; IF did not. The P2Y cartridge had superior sensitivity and specificity to COL/ADP, which was the intention of the manufacturer in the creation of a cartridge more suited to the detection of the clopidogrel effect. This is not surprising from a technical perspective, given that it is less likely that the initial flow rate of blood would be affected by clopidogrel than the other measured values. Although CT is the primary measurand reported by the analyzer, the PHC parameters appear to have equivalent or superior sensitivity and specificity for detection of the clopidogrel effect.

The sensitivity and specificity of the PHC parameters were the highest of the measurands, potentially as they integrate several other values in their calculation and so may provide a more global measure of the clopidogrel effect. For all of the measurands, the values for the COL/ADP cartridge are likely inexact given the very small sample sizes utilized.

PHC1, PHC2, and TV allowed for discrimination of healthy cats from cats with clopidogrel effect, and thus may prove useful as markers of platelet function. They may be useful as an alternative, or in addition to CT, to help elucidate the clopidogrel effect in cases with borderline clopidogrel resistance, or potentially in cases of flow obstruction when a CT is not reported. The PHC parameters provide the most potential as they integrate several other parameters, and appear to perform well in comparison to CT. They also can be calculated in cases of flow obstruction or other errors in analysis, and may hold potential for determining the clopidogrel effect in these situations. IF appears to not be useful as a sole parameter; TV does hold potential for analysis.

The PHC1 and PHC2 values were created to normalize the outputs of the PFA to a percentage scale. This makes the understanding of these values more intuitive to clinicians unfamiliar with these analyzers and potentially more readily interpretable. In human hematology, it is routine to perform mathematical transformations of data for the purpose of standardization; for example, the international normalized ratio (INR) is routinely reported instead of the prothrombin time (PT) for monitoring vitamin K antagonist therapy.^4^ In human and veterinary medicine, coagulation factor quantification is generally reported as a percentage of normal as determined on pooled plasma. Such standardization allows direct comparison of tests performed on different instruments or using different reagents, and also provides a more intuitive understanding of the degree of coagulopathy present. The PHC values may play a similar role for feline platelet function testing in the future.

A significant limitation of our study is the use of different cohorts of cats for the various arms of the study, including both prospective and retrospective groups with different sampling methods. The varied sampling methods may have led to differences in platelet activation and different PFA-200 results between groups. Ideally, the same methodology would have been utilized for all cats to minimize this effect. Methodology for the healthy cats and the clopidogrel group were kept as similar as possible, and a difference would not be expected in these groups. The blood donor group did have a significantly different methodology, which may have led to different results. However, data in humans suggests that needle gauge does not significantly affect hemostatic parameters in cats,^28^ dogs,^9,30^ or humans.^20^ In humans, the use of butterfly catheters did not affect the results of PFA-100 testing compared to direct collection.^17^ These reports suggest that the effects of different collection methods may be less significant than previously thought. In addition, the median CT was not significantly different between the healthy client-owned and blood-donor cats (Suppl. Table 1). Moreover, with respect to clinical monitoring for the clopidogrel effect, the results are effectively binary, within RI indicating no effect, or markedly prolonged (and often no closure) with clopidogrel effect, such that variations associated with the different collection methods are anticipated to have a minimal impact.

A major limitation to any analysis using a PFA instrument is the occurrence of flow obstructions, which occur when flow terminates abruptly or as a result of high initial velocities and occur in up to 37% of feline samples.^13^ Flow obstructions prevented duplicate analysis of some samples in our study; however, the CVs were relatively small, and in no case would a cat have been miscategorized as clopidogrel-responsive or clopidogrel-resistant based on a single sample. Similar CV results are reported in the human literature,^11^ and the values in our study are comparable to previous veterinary reports.^13^ The major benefit of sample collection with intent for duplicate analyses is to ensure that adequate sample is present to correct for a flow obstruction and not to improve reliability of the test.

Another limitation to our study was the sample size available. Given the difficulty in recruiting an adequate number of healthy cats, we limited our study to 20 animals, which is the minimum amount for RI generation.^6^ Although we calculated 90% CIs around the RI, the sample size may be too small for these to be accurate. Further work is likely needed to validate these findings in a larger population of cats. However, previous studies on the PFA have shown RIs for CT for COL/ADP of 44–94 s (median: 69 s)^13^ and 43–176 s (median: 69s).^16^ These RIs compare favorably with our calculated RI of 41–104 s (median: 52 s) and suggest that other results may be similar.

The sample size for our COL/ADP group was very small, and the calculations of sensitivity and specificity may be inaccurate for this group. Given that P2Y testing is considered more accurate for the detection of a clopidogrel effect, COL/ADP is anticipated to be the less relevant parameter; nonetheless, the performance of the assays reported here should be interpreted with caution.

Specificity was determined in a healthy and not diseased population; therefore, this test cannot be considered specific for the clopidogrel effect. In clinical usage, a clinician will be aware that the test is being used in a cat on clopidogrel therapy as a test for its effect.

The TV_median_ value was determined for our analyzer, allowing calculation of PHC parameters. It is likely that this value may vary on different analyzers and in different laboratories, which would require the laboratories to determine their own TV_median_. If this is the case, PHC parameters would be useful as they would allow the comparison of results between analyzers that may have slightly different values for CT, analogous to the calculation of the INR from PT in human medicine.

We found that the PHC1, PHC2, and TV parameters are valid for determining the clopidogrel effect in cats. These parameters allow normalization of CT and potentially direct comparison between cartridges and models of PFA, if a laboratory is able to determine a TV_median_ for its instrument, whereas CT does not allow for this normalization. Although further work is needed to determine additional uses of our research values in various disease states and the occurrence of flow obstruction, they may be used to provide a more intuitive measure of platelet function to practitioners.

## Supplemental Material

sj-pdf-1-vdi-10.1177_10406387231197440 – Supplemental material for Investigation of Platelet Function Analyzer 200 platelet function measurements in healthy cats and cats receiving clopidogrel Click here for additional data file. Supplemental material, sj-pdf-1-vdi-10.1177_10406387231197440 for Investigation of Platelet Function Analyzer 200 platelet function measurements in healthy cats and cats receiving clopidogrel by Matthew R. Kornya, Anthony C. G. Abrams-Ogg, Shauna L. Blois and R. Darren Wood in Journal of Veterinary Diagnostic Investigation
