# Supplementary material for: Investigation of Platelet Function Analyzer 200 platelet function measurements in healthy cats and cats receiving clopidogrel
Source: J Vet Diagn Invest. 2023 Aug 30;35(6):664–70. doi: 10.1177/10406387231197440 (PMC10621536; doi:10.1177/10406387231197440)
Supplement: sj-pdf-1-vdi-10.1177_10406387231197440 – Supplemental material for Investigation of Platelet Function Analyzer 200 platelet function measurements in healthy cats and cats receiving clopidogrel [file sj-pdf-1-vdi-10.1177_10406387231197440.pdf]

Kornya MR, et al. Investigation of Platelet Function Analyzer-200 platelet function measurements in healthy cats and cats receiving clopidogrel

Supplemental Table 1. Median values for healthy cats, blood donors, pooled healthy cats and blood donors, and clopidogrel-treated cats for a variety of measurands.

	Healthy cats (*n* = 20)	Blood donors (COL/ADP *n* = 6; P2Y *n* = 35)	Pooled healthy cats/blood donors	Clopidogrel-treated cats (COL/ADP *n* = 12; P2Y *n* = 21)
COL/ADP				
CT, s	52 (22–81)	62 (46–300)	58 (22–300)	146 (23–268)
IF, µL/min	246 (199–265)	231 (279–176)	243 (199–265	278 (198–343)
TV, µL	273 (240–367)	277 (248–549)	274 (240–549)	443 (128–758)
PHC1, %	94 (52–136)	102 (28–132)	101 (28–136)	38 (15–74)
PHC2, %	99 (94–105)	100 (60–103)	100.0 (60–105)	87 (55–97)
P2Y				
CT, s	51 (11–90)	58 (43–124)	56 (11–124)	300 (211–300)
IF, µL/min	166 (110–211)	215 (154–269)	196 (110–269)	180 (129–255)
TV, µL	211 (182–320)	249 (214–366)	235 (182–366)	631 (435–869)
PHC1, %	88 (22–154)	100 (47–149)	99 (22–154)	14 (9–23)
PHC2, %	99 (90–108)	100 (89–104)	100 (89–108)	49 (20–72)

Reported as median (min.–max.). Normality was assessed using the Shapiro–Wilk test. Normal data were compared with the Student *t*-test, and non-normal data with the Mann–Whitney U test. IF was normally distributed; however, CT and TV were not. For

COL/ADP, none of CT, IF, or TV were significantly different between client-owned healthy cats and blood donors ($p = 0.130$, $p = 0.258$, and $p = 0.247$, respectively). For P2Y, CT was not significantly different between client-owned healthy cats and blood donors ($p = 0.070$), but IF and TV were significantly different between these groups ($p < 0.001$ for both parameters).
